# Design and Evaluation of a Crosslinked Chitosan-Based Scaffold Containing Hyaluronic Acid for Articular Cartilage Reconstruction

**DOI:** 10.3390/molecules30102202

**Published:** 2025-05-17

**Authors:** Salim Hamidi, Mickael Maton, Feng Hildebrand, Valérie Gaucher, Cédric Bossard, Frédéric Cazaux, Jean Noel Staelens, Nicolas Blanchemain, Bernard Martel

**Affiliations:** 1 Univ. Lille, INSERM, CHU Lille, U1008-Advanced Drug Delivery Systems and Biomaterials, F-59000 Lille, France; hamidisalim46@gmail.com (S.H.); mickael.maton@univ-lille.fr (M.M.); feng.hildebrand@univ-lille.fr (F.H.); cedric.bossard@univ-lille.fr (C.B.); nicolas.blanchemain@univ-lille.fr (N.B.); 2 Univ. Lille, CNRS, INRAE, Centrale Lille, UMR 8207—UMET—Unité Matériaux et Transformations, F-59000 Lille, France; valerie.gaucher@univ-lille.fr (V.G.); frederic.cazaux@univ-lille.fr (F.C.); jean-noel.staelens@univ-lille.fr (J.N.S.)

**Keywords:** polymeric scaffolds, chitosan, hyaluronic acid, crosslinking hydrogels, cartilage tissue engineering, porous morphology, mechanical stability, enzymatic degradation, drug release, ciprofloxacin

## Abstract

Polymeric scaffolds are promising in tissue engineering due to their structural similarity to extracellular matrix components. This study aimed to design freeze-dried hydrogels based on chitosan (CHT) and hyaluronic acid (HA). Chitosan-based gels were crosslinked with oxidized maltodextrin (MDo) before the freeze-drying step, resulting in spongy porous scaffolds. Based on the state-of-the-art, our hypothesis was that crosslinking would increase scaffold stiffness and delay the degradation of the CHT:HA resorbable scaffolds swelled in a hydrated physiological environment. The physicochemical and mechanical properties of crosslinked CHT- and CHT:HA-based scaffolds were analyzed. Hygroscopic and swelling behavior were assessed using dynamic vapor sorption analysis and batch studies. Degradation was evaluated under different conditions, including in phosphate-buffered saline (PBS), PBS with lysozyme, and lactic acid solutions, to investigate scaffold resistance against enzymatic and acidic degradation. The porosity of the spongy materials was characterized using scanning electron microscopy, while dynamic mechanical analysis provided information on the mechanical properties. Crosslinked scaffolds showed reduced swelling, slower degradation rates, and increased stiffness, confirming MDo as an effective crosslinking agent. Scaffolds loaded with ciprofloxacin (CFX) demonstrated their ability to deliver therapeutic agents, as the CFX loading capacity was promoted by CHT–CFX interactions. Microbiologic investigation confirmed the results. Finally, cytotoxicity tests displayed no toxicity. In conclusion, MDo-crosslinked CHT and CHT:HA scaffolds exhibit enhanced stability, functionality, and mechanical performance, making them promising for cartilage tissue engineering.

## 1. Introduction

Articular cartilage or hyaline cartilage is a connective tissue covering the joint surfaces of bones, and it plays a key role in supporting joint movement by absorbing loads and reducing friction [1]. Structurally, hyaline cartilage is an anisotropic tissue organized into three different zones with their own structural and functional organization: the superficial, middle, and deep zones [2]. Biologically, cartilage is supplied by a single cell source with a limited number; indeed, chondrocytes are available in 5% to 10% of cartilage tissue [2]. They play a key role in the homeostasis of cartilage, and allow the biosynthesis of the different essential elements of the extracellular matrix (ECM), such as type II collagen, sulfated glycosaminoglycans (GAGs), and other molecules, responsible for the mechanical behavior of cartilage [2].

Once damaged, cartilage has a poor capacity for self-regenerating because of its avascularity and low cellular bioactivity [3]. Consequently, this can result in chronic disease like osteoarthritis, which affects the aged population over 60 years old with a predominance for women of 18%, and for men of more than 9% [4]. The disease evolves in a complex pathological context, affecting the whole joint including the cartilage, subchondral bone, and synovium; however, it is well recognized that early cartilage degradation and the alteration of the underlying subchondral bone play an active role in the disease pathogenesis [4,5]. According to the International Cartilage Regeneration and Joint Preservation Society (ICRS) [6], osteochondral defects are classified into two different types depending on their depth, as partial or full thickness lesions. Based on this classification, different clinical approaches have been developed, including palliative, reparative, and regenerative treatments [4].

Palliative treatments, such as arthroscopic debridement and chondroplasty, were aimed at reducing the inflammatory response and pain to improve patient function. These techniques have been gradually abandoned in recent decades due to the lack of real benefit and the potential presence of adverse effects [4]. The surgical approach results in the formation of fibrocartilaginous tissue, which is known to differ in terms of histological, biomechanical, and viscoelastic properties. Subsequently, a regenerative cell biological method was developed by Dr Peterson and Dr Brittberg, consisting of autologous chondrocyte implantation (ACI). However, the main challenge associated with this technique is the maintenance of the chondrocyte phenotype over time [4]. In fact, chondrocytes cultured in a two-dimensional environment may dedifferentiate into fibroblasts, leading to the production of fibrous tissue that is completely different from native hyaline cartilage. Therefore, ACI has been improved by the preliminary in vitro proliferation of cells supported on a porcine collagen matrix before transplantation into the defect site [4].

Recent studies have explored a variety of innovative approaches for osteochondral tissue engineering. For example, multiphasic scaffolds composed of alginate and gelatin—fabricated via 3D printing and enriched with different concentrations of platelet-rich plasma (PRP)—have demonstrated that a 2% PRP loading markedly enhances the chondrogenic differentiation of bone marrow-derived mesenchymal stem cells, as well as the synthesis of extracellular cartilage matrix components [7]. Another study by P. Guan et al. designed exosomes derived from bone marrow mesenchymal stem cells loaded with an ECM-biomimetic hydrogel. The findings demonstrated that this hydrogel effectively stimulated chondrocyte anabolic activity by modulating and reducing the inflammatory response [8]. Among the recent advances, gene therapy has gained attention for its ability to support cartilage repair by continuously delivering therapeutic genes. In this regard, gene-activated matrices (GAMs) offer a promising solution. For instance, by embedding gene complexes directly into bioengineered scaffolds, they allow for a more sustained and localized release of these genes, creating a favorable environment for long-term cartilage regeneration [9]. Another strategy using a cell-free biofunctionalized scaffold described a potential alternative to promote cartilage regeneration. Indeed, Z. Mao et al. described the use of silk fibroin loaded with two bioactive molecules: (1) E7 peptide, known for its affinity to bone marrow mesenchymal stem cells (BMSCs), and (2) transforming growth factor (TGF-β1). In vivo implantation of this implant in a rabbit model showed in situ cartilage regeneration [10]. Another approach consisted of interpenetrated network hydrogel-based β-cyclodextrin-modified alginate/decellularized cartilage ECM reinforced with a 3D-printed PCL/starch microfiber as a carrier for drug loading like kartogenin. This construction was considered to be a promising alternative in term of mechanics and biological performance for cartilage tissue engineering [11].

The emergence of tissue engineering has gained growing interest as a potential solution to restore damaged tissue. The principle of this concept is based on the use of cells, scaffolds, and signaling factors, alone or in combination [2], to mimic the native tissue and to offer a favorable microenvironment for complex tissue reconstruction [4]. The scaffold for cartilage tissue engineering should have some essential requirements, like promoting cell adhesion, proliferation, and ECM production, through the three-dimensional structure. The porosity and pore size are important parameters to enable nutrient diffusion and waste exchange, in addition to integrity and sufficient mechanical properties with controllable hydrolytic and enzymatic degradation, which are highly suitable for the ideal scaffold [2].

Polymeric materials are commonly used in biomedical applications because of their availability and structural similarity to ECM tissue [12]. In cartilage tissue engineering (CTE), natural polymers including polysaccharides, glycosaminoglycans, and proteins are widely used, whereas in the synthetic polymers range, polyesters like PLGA poly(lactic-co-glycolic acid) are frequently selected [12]. Polysaccharides such as agarose, starch, alginate, and chitosan are very attractive; indeed, R M. Jeuken et al. described the structural similarity to native GAGs of these biomaterials, and the hydrated nature of these polymers allows the distribution of mechanical force and nutrient exchange [12].

Chitosan (CHT) is a promising polysaccharide for scaffold design, as it is one of the rare natural polymers to be positively charged in an acidic medium [13]. CHT is derived from chitin deacetylation and composed of β 1-4-linked 2-amino-2-deoxy-D-glucose. CHT is commonly used in biomedical and pharmaceutical fields for its biocompatibility, biodegradability, antibacterial, and antifungal activities and also its drug delivery property [14]. In addition, it shares a structural analogy with GAGs of articular cartilage [15].

The physicochemical and mechanical properties of CHT could be tailored and enhanced by combining it with a synthetic polymer like polycaprolactone (PCL) or a natural polymer like hyaluronic acid (HA) [16]. HA presents in the tissues of living organisms [13]. It is an essential component of the cartilage ECM, and HA plays a favorable role in chondrocyte behavior by enhancing cellular function through a signaling pathway [3]. HA is a negatively charged polymer, capable of interacting with other positively charged polymers such as chitosan to form polyelectrolyte complexes (PECs) [13]. In particular, Correia et al. also combined CHT and HA for preparation of freeze-dried scaffolds designed for cartilage tissue engineering [3]. H. Tan et al. developed an injectable composite hydrogel by combining *N*-succinyl-chitosan and oxidized hyaluronic acid. In this later study, the gelation process was attributed to Schiff base formation between both chemically modified polymers, and the study demonstrated that this hydrogel could effectively encapsulate and deliver cells like articular chondrocytes, indicating its potential in cartilage tissue engineering [17]. Nath et al. developed a CHT-HA polyelectrolyte complex crosslinked with genipin for the immobilization of BMP-2 growth factor for bone and soft tissue regeneration [18]. S. Escalante et al. developed a CHT-HA hydrogel chemically crosslinked with a diisocyanate and loaded with chondroitin sulphate (CS) that displayed a good potential in the improvement of blood clot stabilization during microfracture surgery, a surgical technique promoting cartilage tissue regeneration [19]. In all these studies involving CHT-HA blends applied to hydrogel scaffolds for cartilage regeneration, the authors observed that modulating the CHT/HA ratio and varying the density of chemical crosslinks in the hydrogels allowed control of the pore size, swelling ratio, degradation, interactions with bioactive compounds (growth factors and drugs), release kinetics, cell adhesion (osteoblasts and chondrocytes), and mechanical properties of the resulting scaffolds.

Indeed, chitosan, through its functional reactive groups like hydroxyl and amino groups, can be chemically crosslinked by covalent bonding, or physically crosslinked by ionic interactions through its protonated amino groups; in addition, weak polymer–polymer interactions can also occur through hydrogen bridges or through hydrophobic interactions and van der Waals bonding [20]. Different low molecular weight crosslinking agents have been described in the literature, and the most well-known are formaldehyde [21], glutaraldehyde [22], genipin [23], and epoxy components [24]. These products have demonstrated their efficacy, particularly by significantly improving the mechanical properties of scaffold-based chitosan. However, a cytotoxic effect of such low molecular weight crosslinkers is usually observed [20]. Dialdehyde starch can be defined as an aldehyde-rich component which is produced from starch oxidation by sodium iodate [25]. It is used as a crosslinking agent of polysaccharides and proteins in drug carriers and in scaffolds for tissue engineering [20,26] due to its biodegradability and acceptable cytotoxicity compared with its aldehydic low molecular weight analogs, especially formaldehyde and glutaraldehyde, as mentioned above [27].

Therefore, the goal of this study was to prepare a 3D porous scaffold based on biopolymers such as CHT and HA, known to mimic the cartilage ECM, and provide a favorable environment to induce cartilage tissue engineering. Interestingly, maltodextrin, a short chain oligosaccharide issued from starch, was oxidized and used as a crosslinker of CHT in order to enhance the stability and delay the biodegradation of highly hydrophilic porous scaffolds issued from the freeze-drying of CHT-HA-based solutions. Our hypothesis was that the crosslinking reaction would enhance the mechanical properties of CHT:HA scaffolds, with the objective of reaching the structural properties required for application in cartilage regeneration. Therefore, crosslinking’s influence on the swelling properties, porosity, stability in aqueous media, and mechanical properties of scaffolds was investigated. Additionally, another challenge here was to study the possibility of liberating a drug in a prolonged time by scaffolds. Therefore, the adsorption, release profile, and antimicrobial properties of ciprofloxacin-loaded scaffolds were finally investigated.

## 2. Results and Discussion

### 2.1. FTIR Analysis

**Figure 1:**
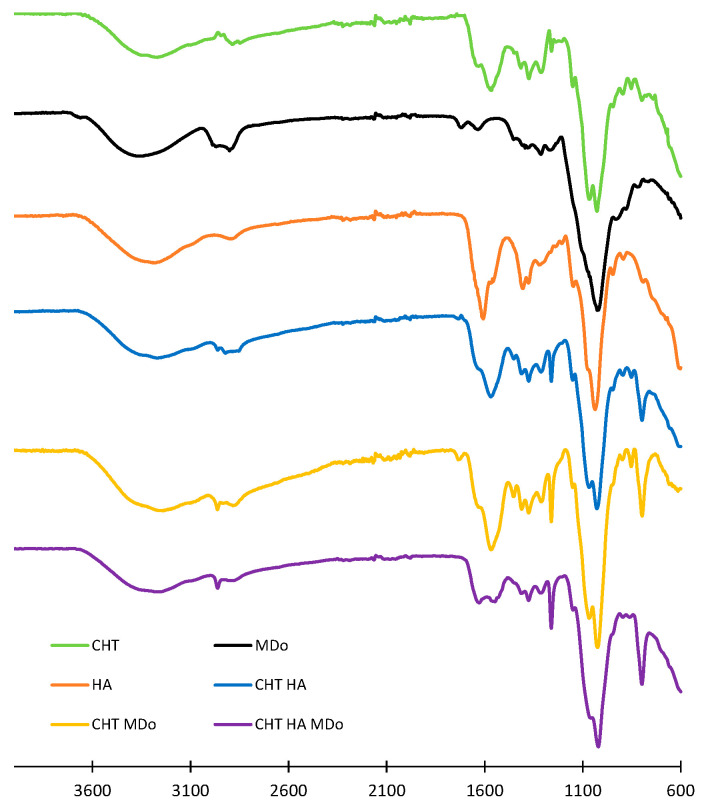
ATR-FTIR spectra of the sponges CHT, CHT:HA, CHT:MDo, and CHT:HA:MDo, and spectra of the MDo and HA powders.

The CHT, HA, CHT:HA, CHT:MDo, and CHT:HA:MDo scaffolds detailed compositions are displayed in Table 1. They were analyzed by ATR-FTIR (spectra shown in Figure 1). The spectrum of the CHT sponge displays wide bands above 3000 cm^−1^ corresponding to the stretching vibration of OH and –NH_2_ groups, while the band at 2885 cm^−1^ is associated to the stretching of C–H bonds. The band at 1566 cm^−1^ corresponds to the bending of an N–H bond (amide II) and 1310 cm^−1^ to C–N stretching (amide III). The band at 1375 cm^−1^ corresponds to the deformation of CH_3_ and that at 1027 cm^−1^ corresponds to the C–O bond elongation [28]. The band at 1259 cm^−1^ corresponds to the deformation vibration of carboxylic acid (C–OH), and the peak at 797 cm^−1^ to the deformation vibration of C–H.

Interestingly, this band at 797 cm^−1^ was not observed in the CHT raw powder form but appeared in the CHT sponge spectrum with a low intensity, and presented a stronger intensity in the spectra of sponges made of CHT blended with HA and MDo. Such a remark is also valid for the band at 1259 cm^−1^. Such features can be attributed to the increased crystallinity of CHT in the lyophilized scaffolds.

In the spectrum of MDo, the band at 1716 cm^−1^ is associated with the stretching of the C=O aldehyde group. This evidences the oxidation of MD into MDo [29,30,31,32]. This band is not present in the spectra of CHT sponges, suggesting the full conversion of the aldehyde groups into imine bonds upon the reaction of MDo with CHT [30]. The HA spectrum displays characteristic absorption bands, including a broad peak at 3327 cm^−1^ attributed to the –OH stretching vibration. The peak at 2876 cm^−1^ corresponds to the CH_2_ stretching vibration. Bands at 1610 cm^−1^ and 1403 cm^−1^ are associated with the symmetric and asymmetric stretching vibrations of the COO^−^ group, respectively. A band observed at 1034 cm^−1^ also relates to the C–O–C stretching vibration within the saccharide repeat units of HA [19,33,34]. In the CHT:HA and CHT:HA:MDo spectra, the absence of the bands at 1610 and 1403 cm^−1^ suggests polyelectrolyte complex formation between CHT and HA [19]. Some authors reported peak positions of C=N in the Schiff base nearby at 1636 cm^−1^ [20,25,29], while others reported the detection of characteristic peaks between 1610 and 1665 cm^−1^ [35,36]; moreover, R. Tang et al. reported the formation of the Schiff base at 1653 cm^−1^ [25]. In our study, the C=N vibration bands expected in the spectra of CHT:MDo and CHT:HA:MDo were not clearly located due to the low crosslink density in both formulations, containing 5% CHT and only 0.4% MDo. Additionally, A. Serrero et al. reported that imine moieties are difficult to characterize because of the reversible nature of this bond. In another consideration, the FTIR signal of the C=N bond could also be masked by multiple peaks such as the amide group of CHT [30].

### 2.2. Swelling Study of Sponges

**Figure 2:**
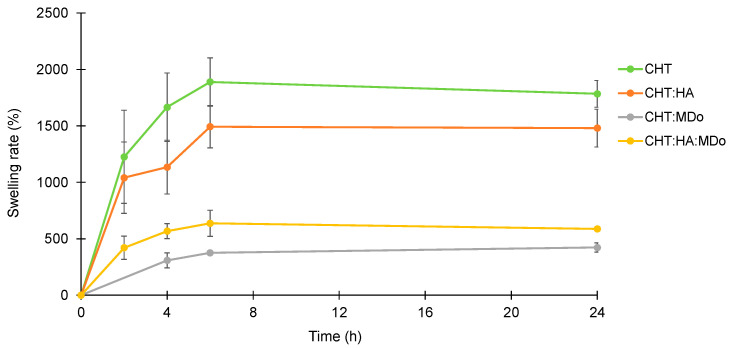
Swelling profiles of the CHT, CHT:HA, CHT:MDo, and CHT:HA:MDo sponges incubated for 24 h in PBS medium (37 °C, 80 rpm), n = 3.

Figure 2 shows the swelling kinetics of the sponges in PBS (pH = 7.4). The experiment was conducted at 37 °C under agitation at 80 rpm for different incubation periods (2 h, 4 h, 6 h, and 24 h). The mass of the wet sponges was continuously monitored throughout the incubation. The results are expressed in term of the swelling rate, calculated from Equation (1). The PBS uptake of the sponges increased during the first 6 h of immersion, and then a plateau was reached, as observed by Y. Deng et al. with a CHT:HA hydrogel [37]. Table 2 displays the swelling rates of all sponges measured at 24 h.

Overall, CHT and CHT:HA sponges showed a greater swelling rate compared to chemically crosslinked groups containing MDo. After 24 h of incubation, the swelling ratios (reported in Table 1) were 1785 ± 119% for CHT and 1480 ± 166% for CHT:HA, whereas the chemically crosslinked groups showed lower values: 423 ± 46.8% for CHT:MDo and 587 ± 10.9% for CHT:HA:MDo (Table 2). This reduction in swelling can be attributed to the chemical crosslinking of CHT by MDo, which leads to the formation of a covalent network that reduces polymer chain mobility.

A similar trend was observed between CHT and CHT:HA. Although HA is known for its high hygroscopicity, its electrostatic interactions with CHT reduced the chains’ mobility by these physical crosslinks and consequently reduced its water absorption capacity compared with CHT sponges. In contrast, the presence of HA in the CHT:HA:MDo system enhanced the water absorption capacity (+28%) compared with CHT:MDo. This suggests that the crosslinking of CHT by MDo was not affected by the presence of HA, but the hydrophilic groups of HA contribute to enhance the water retention of the system.

The swelling behavior of CHT:HA sponges can be attributed to the presence of hydrophilic functional groups, such as carboxyl, amine, and hydroxyl groups, in the polymer structures of CHT and HA. The initial rapid and high water uptake was mainly due to the hydrophilic character of CHT and CHT:HA and to the porous structure of the sponges.

The water uptake capacity of the scaffold is an essential parameter for cell adhesion and proliferation, and simulates the interaction with blood during surgical procedures like microfracture. According to S.Escalante et al., the absorption mechanism needs to be faster to stabilize the blood clot while allowing it to be loaded with growth factors and cells derived from the clot [19].

The high swelling capacity of the non-crosslinked sponges (CHT alone and CHT:HA) was attributed to the water affinity of CHT and HA, namely, the presence of hydrophilic groups like the carboxylate groups of HA, protonated amino groups of CHT, and hydroxyl groups in both polysaccharidic structures. Furthermore, the initial high water absorption rate by the sponges (plateaus reached from 6 h) was due to the macroporous structure of the sponges that allowed the scaffolds to rapidly absorb the PBS solution by capillarity, filling the porous volume. Compared with the CHT sponges, the CHT:HA samples displayed a reduced swelling capacity due to the formation of ionic interactions between both polyelectrolytes that reduced the macromolecular mobility in the system. Additionally, scaffold chemical crosslinking with MDo decreased swelling capacity by a larger extent, as observed for CHT:MDo and CHT:HA:MDo.

This could be explained by the formation of covalent crosslinks resulting in a polymeric network with reduced mobility [38]. The same phenomenon was observed by S.D. Nath et al., who reported elsewhere that crosslinking of the CHT:HA system with different concentrations of genipin resulted in a reduced swelling ratio in PBS [18]. This swelling study was then completed by a SEM investigation in order to understand the consequences of an immersion of scaffolds in a physiologic medium on the porous structure of all studied sponges’ groups.

### 2.3. SEM Analysis

**Figure 3:**
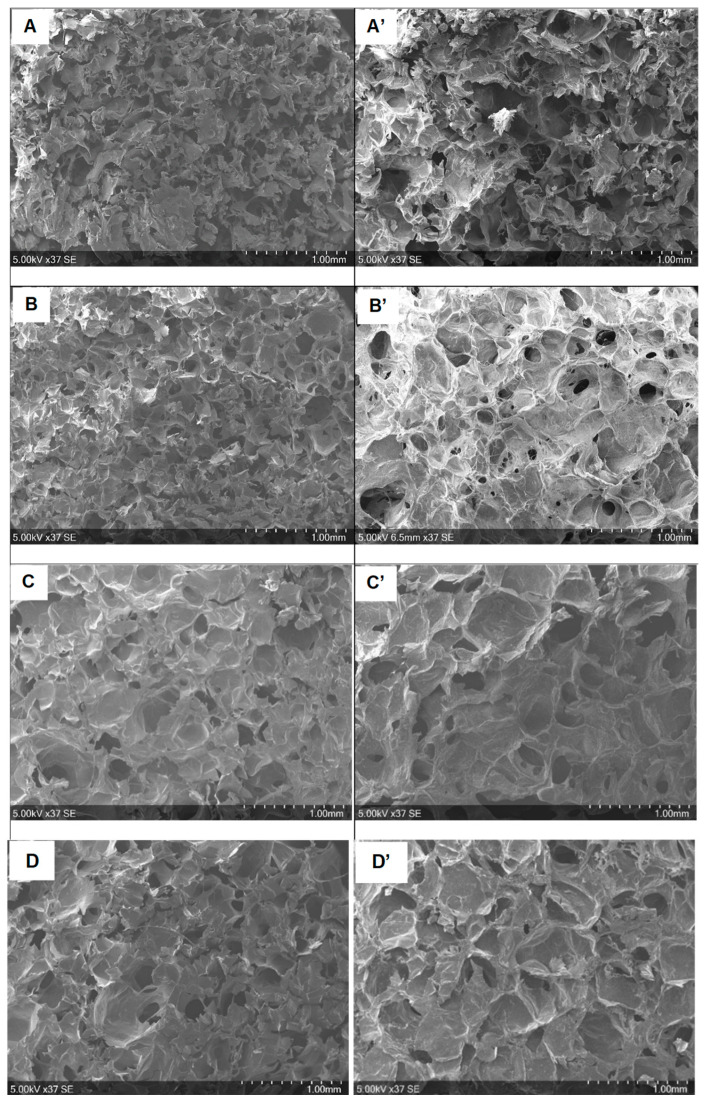
SEM micrographs corresponding to different groups before (**A**–**D**) and after (**A’**–**D’**) swelling for 24 h in PBS. (**A**,**A’**): CHT; (**B**,**B’**): CHT:HA; (**C**,**C’**): CHT:MDo; (**D**,**D’**): CHT:HA:MDo.

Figure 3 presents SEM micrographs of the cross-sections of the sponges, which reveal an interconnected porous structure with heterogeneity in terms of distribution and pore size. This variation depended on the sponge composition, as detailed in Table 1.

The non-crosslinked CHT and CHT:HA scaffolds (Figure 3A,B) displayed smaller pore sizes than their MDo-crosslinked counterparts (Figure 3C,D). After a first SEM analysis, the scaffolds were then swelled for 24 h in PBS, freeze-dried, and analyzed again by SEM. Pore size expansion following swelling was observed by comparing Figure 3A–D with Figure 3A’–D’. In particular, pore size extension was more pronounced after this treatment for samples not crosslinked with MDo, with an extension of +63% for CHT and +27.8% for CHT:HA, compared with +2.6% for CHT:MDo and +4.4% for CHT:HA:MDo samples after the swelling–freeze-drying cycle. The extension of the pore size after sponge hydration in PBS was correlated with the measured swelling ratios discussed in the previous section. The results confirm that sponge swelling depends on the capacity of pores walls to stretch. Thus, in the case of non-crosslinked sponges, the pore volume increased extensively and residual deformation of pore walls was observed in dried sponges due to the creeping phenomenon in the polymeric networks; on the contrary, in the case of MDo-crosslinked systems, pore swelling was reduced due to the reduction of the creeping phenomenon caused by covalent crosslinks, resulting in reduced residual deformation of the porous structure after the swelling–drying cycle. The pore size range of our scaffolds (200–300 µm) differs from the literature data, i.e., 10–100 µm (Tan et al. [17]), 50–90 µm (Nath et al. [18]), and 77–97 µm (Correia et al. [3]). Such differences may be attributed to the different scaffold preparation protocols, such as polymer concentrations in the precursor hydrogel, temperature of freezing before freeze-drying, etc. It is worth mentioning that our SEM analyses were focused on the center of the cross-sections of the cylindrical samples, and that a rim effect was observed as the pore size lowered, and observations focused from the center toward the edge of scaffolds. However, the above-cited reference works mention that such open-pore structures of dimensions below 100 µm are appropriate for metabolite circulation and cellular infiltration in the scaffolds. Therefore, our scaffolds, with around twice larger pore sizes, should also present similar properties.

### 2.4. DVS Analysis

**Figure 4:**
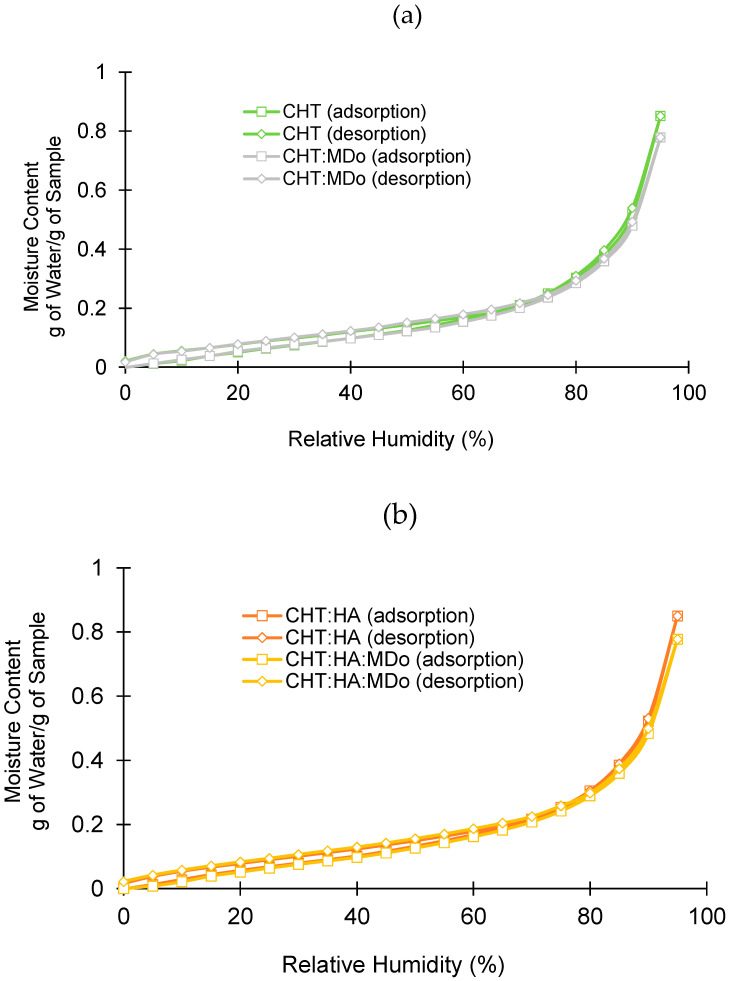
Dynamic vapor sorption analysis: (**a**) CHT and CHT:MDo; (**b**) CHT:HA and CHT:HA:MDo.

Figure 4 shows the different adsorption and desorption profiles of all sponge groups as a function of relative humidity (RH). In all cases, the sorption and desorption profiles were similar, without hysteresis.

In adsorption and desorption cycles in the 0–70% RH domain, water adsorption was moderate, unlike in the range of 80–95% RH, where the dependence of water adsorption on RH was more marked. The water adsorption of sponges at 95% RH is displayed in Table 2. Two groups are distinguishable: both non-crosslinked sponges on the one hand (0.85 g H_2_O/g), and crosslinked sponges on the other hand (0.77–0.78 g H_2_O/g for CHT:MDo and CHT:HA:MDo, respectively). The reduction of the hygroscopic character of the scaffolds upon crosslinking can be explained by the reduction of the hydrophilicity of the materials’ surfaces caused by the reduction of amino groups of CHT involved in the reaction with aldehydes of MDo. Therefore, the hygroscopic character of scaffolds measured by DVS evolves in parallel with their swelling capacity, as mentioned in Section 2.2. Indeed, these results differ from those obtained in the swelling test because water vapor adsorption by the material surface and bulk liquid water absorption in the porous volume of the scaffold are fundamentally different phenomena. According to S. Rastogi et al., adsorption involves water molecules interacting with the material’s surface, while absorption occurs when water penetrates the material’s bulk [39].

### 2.5. In Vitro Degradation Studies

The sponges were immersed in different aqueous media in order to observe their stability (i) at physiological pH (PBS 7.4), (ii) in the presence of lysozyme (selectively degrading CHT), and (iii) in an acidic medium. The remaining weight, expressed in percent, was calculated according to Equation (2).

**Figure 5:**
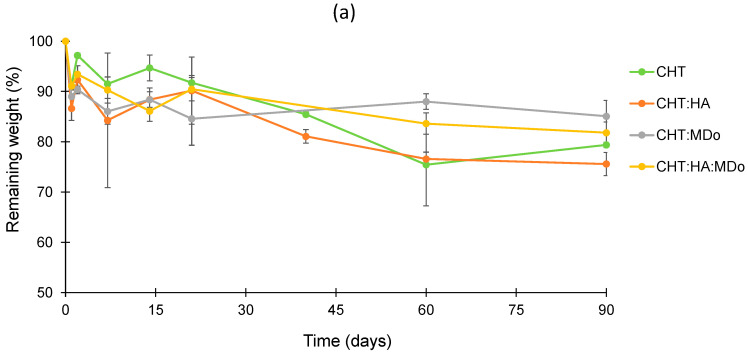

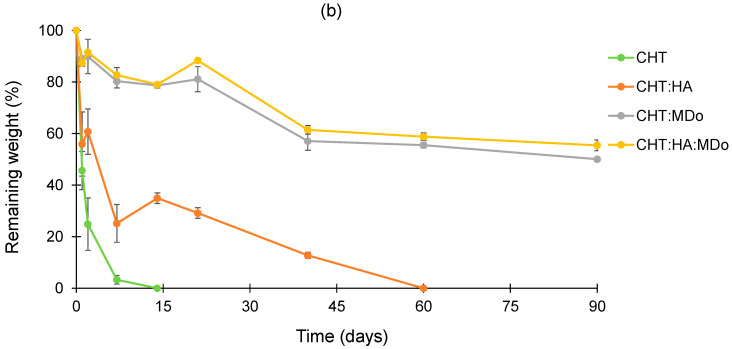
In vitro degradation profiles of CHT, CHT:HA, CHT:MDo, and CHT:HA:MDo sponges during 90 days (**a**) in PBS at pH 7.4, and (**b**) in PBS at pH 7.4 enriched with lysozyme 0.5 mg/mL. T = 37 °C, agitation at 80 rpm (n = 3).

Within the degradation period of 90 days, the plots in Figure 5a presented slow and limited degradation of all sponges in PBS. After 90 days, the weight loss values measured for CHT:MDo and CHT:HA:MDo were, respectively, 14.9 ± 3.1% and 18.2 ± 2.1%, against 20.6 ± 0.5% and 24.4 ± 2.3% for CHT and CHT:HA, respectively.

In contrast, in Figure 5b, the degradation kinetics in enzymatic solutions revealed a mass loss occurring from the first days of incubation, particularly pronounced for the CHT and CHT:HA sponges, with respective decreases of 54 ± 7% and 44 ± 12%. The degradation of CHT sponges continues at a faster rate, and was complete after 14 days. Similarly, the CHT:HA sponges displayed a slower degradation profile, with a degradation of 65 ± 2% after 14 days, ultimately reaching complete degradation by day 60.

The sponges crosslinked with MDo exhibited a slower enzymatic degradation rate. As a matter of fact, the CHT:MDo and CHT:HA:MDo sponges showed degradation extensions during the first 21 days of 19 ± 5% and 11.6 ± 1%, respectively, and reached 50 ± 1% and 44.5 ± 2% after 90 days. Therefore, sponge crosslinking with MDo caused resistance toward enzymatic degradation and prolonged the life of scaffolds over 3 months.

These results suggest that the covalent network formed after crosslinking with MDo enhances the stability of CHT and CHT:HA systems in an enzymatic environment. This extended stability is important to support cartilage regeneration through chondrocyte proliferation and ECM synthesis, with the material gradually degrading over time, thereby enabling the formation of a new tissue [40]. Similarly, Long Bi et al. reported in their study that crosslinking a chitosan/collagen scaffold with different concentrations of genipin led to a reduction in degradation rates in an enzymatic environment [41]. On the other hand, crosslinking CHT with glutaraldehyde or oxidized dextran produced a porous scaffold with enhanced stability and mechanical properties; additionally, crosslinking CHT with glutaraldehyde was shown to promote chondrocyte proliferation [42]

According to J.A. Jennings et al., enzymatic degradation is the primary degradation mechanism of CHT in vivo. Lysozyme degrades CHT chains by cleavage of the β (1–4) glycosidic bonds linking the *N*-acetylglucosamine residue in polysaccharide units [43].

The degradation rate of a CHT-based scaffold is an important parameter for the tissue engineering field, and has to match the requirement of slow degradation to maintain satisfactory mechanical properties and enable the support of tissue regeneration [44]. In our study, CHT and CHT:HA crosslinked with MDo displayed slower degradation in enzymatic media compared with control groups, because the crosslinking density reduced the penetration of lysozyme into the CHT bulk and slowed degradation [45]. In addition, according to Y. Privar et al., the enzymatic degradation of CHT is influenced by its crystallinity and deacetylation degree [46].

**Figure 6:**
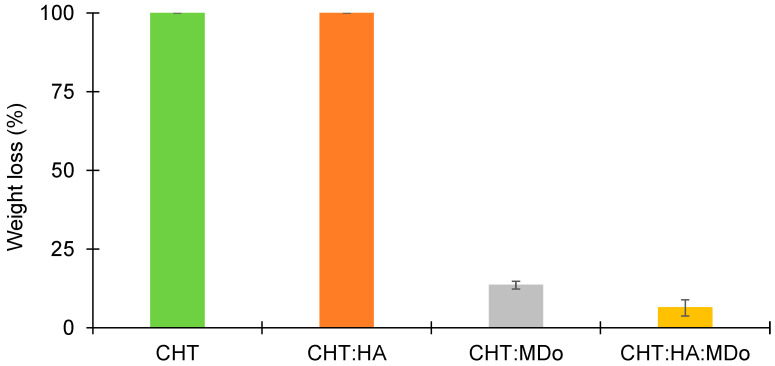
Weight loss of sponges CHT, CHT:HA, CHT:MDo, and CHT:HA:MDo after 7 days of incubation in 1% *v*/*v* lactic acid solution (n = 3).

The stability of different sponges was assessed under acidic conditions (pH = 2.3). The results are presented in Figure 6, and show that both the CHT and CHT:HA sponges are completely dissolved (100%) after 7 days of incubation. However, the MDo-crosslinked sponges presented lower mass losses, of only 13.5 ± 1.3% for CHT:MDo and 6.4 ± 2.6% for CHT:HA:MDo (Figure 6). The findings show that the crosslinking of CHT and MDo stabilizes the sponges under acidic conditions. The reason for the rapid enzymatic degradation of non-crosslinked sponges lies in the higher polymeric network swelling, which facilitates the access of the enzyme to the CHT chains, as compared to the crosslinked systems. Moreover, such conditions may present in the chronic inflammatory process caused by macrophage operation [41]. In fact, activated macrophages release pro-inflammatory cytokines and reactive oxygen species (ROS). Therefore, increased glycolytic activity causes lactate accumulation, resulting in a reduction of tissue pH, establishing an acidic environment.

### 2.6. Dynamic Mechanical Analysis (DMA)

**Figure 7:**
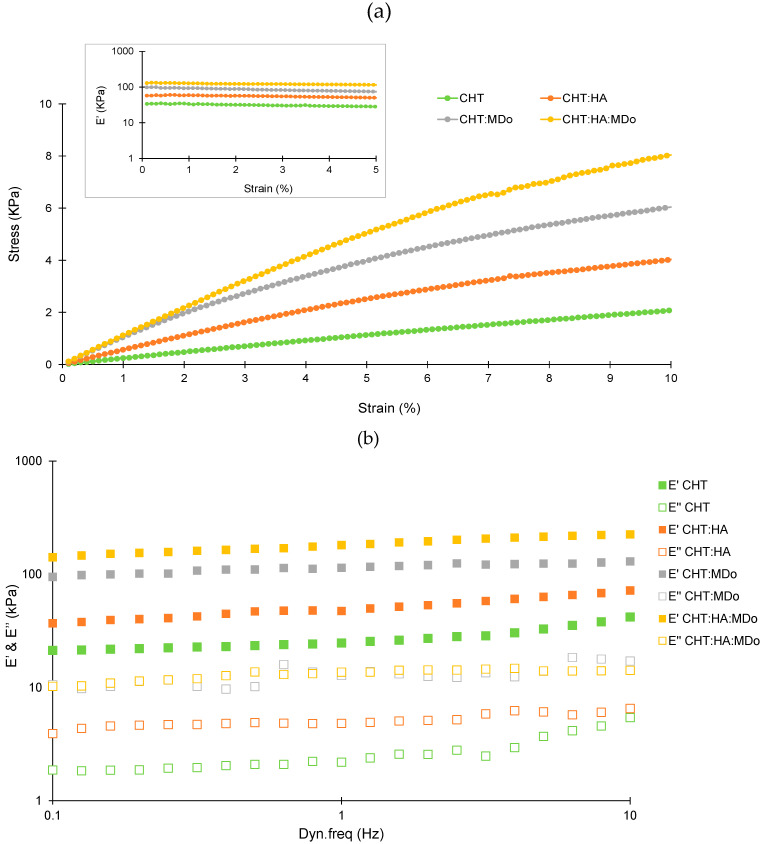
(**a**) Stress–strain sweep test (up to 10% strain) for the different sponges at a 1 Hz oscillation frequency. The test was conducted at 37 °C on wet sponges equilibrated for 24 h in PBS solution. (**b**) Frequency sweep test (0.1–10 Hz) at a controlled 1% strain, where the viscoelastic moduli were measured: the storage modulus (E’) and loss modulus (E’’). The test was performed in triplicate (n = 3). All tests were conducted on wet sponges equilibrated in PBS solution for 24 h at 37 °C.

Figure 7a presents the evolution of stress as the function of strain for the four scaffolds. A considerable improvement of the stress level was observed for the CHT:HA:MDo and CHT:MDo samples, compared with the two control samples without MDo. This result showed an increase of the stiffness with the crosslinking, as expected. Considering that all materials display a linear viscoelastic behavior up to 5% of strain, the evolution of the viscoelastic response has been studied as a function of the dynamic frequency in the linear domain (strain amplitude of 1%) (Figure 7b). The frequency range from 0.1 to 10 Hz was chosen to mimic the mechanical conditions of articular cartilage during normal function. This approach allowed a more physiologically relevant analysis of the mechanical behavior of the sponges under dynamic loading. For all samples, the storage modulus (E’) was higher than the loss modulus (E’’), indicating that the elastic response (solid-like) of the sponges dominates their viscous (fluid-like) behavior over the entire frequency range studied. The storage modulus values (E’) recorded at 1 Hz for the four sponges were 29 ± 4 kPa (CHT), 54 ± 16 kPa (CHT:HA), 145 ± 34 kPa (CHT:MDo), and 197 ± 18 kPa (CHT:HA:MDo). A marked increase in E’ occurs with the crosslinking of CHT by MDo; an enhancement of almost 400% was observed for CHT:MDo and 260% for CHT:HA:MDo compared with their counterparts without MDo. Notably, a smaller increase of E’ was observed between CHT and CHT:HA (86% of enhancement).

Considering CHT sponges as the reference, the increase of E’ values observed above revealed a sponge stiffness enhancement that can be attributed, on the one hand, to ionic interactions between CHT and HA forming a polyelectrolyte complex, and in a more pronounced manner, to MDo covalent crosslinks. Thus, both MDo and HA play complementary roles in enhancing the mechanical properties of the sponges, with MDo primarily contributing to covalent network formation and HA providing additional physical reinforcement through polymer–polymer interactions.

Various studies on crosslinked biopolymers have explored their applications in articular cartilage tissue engineering. A. Subramanian et al. reported that chondrocytes have the ability to sense their mechanical environment, with scaffold stiffness acting as an external stimulus that influences cell proliferation and the preservation of chondrocyte morphology. In their study, chitosan films were crosslinked with diepoxide (1,4-butanediol diglycidyl ether) at varying ratios. It was demonstrated that the chitosan film with the highest crosslinker content (1:5 ratio) exhibited the highest elastic modulus (19.9 kPa), which in turn promoted enhanced chondrocyte growth and biosynthesis activity [47].

Le-Ping Yan et al. conducted a dynamic mechanical analysis (DMA) of collagen/chitosan scaffolds crosslinked with different genipin concentrations (0.1%, 0.3%, and 0.5%). For scaffolds containing 50% chitosan (Coll/CHT-50), they observed that the elastic modulus (E’) increased as the concentration of genipin increased. Specifically, at a frequency of 1 Hz, E’ ranged from approximately 300 kPa to 700 kPa as the genipin concentration was raised from 0.1% to 0.5%, indicating a stiffer material. These values are relatively higher than our results, likely due to the double crosslinking reaction between genipin and both chitosan and collagen, resulting in an interpenetrated network (IPN). From a biological perspective, the crosslinked collagen/chitosan scaffold enhanced chondrocyte attachment and viability [48].

Studies have investigated the impact of crosslinking and stiffness in biopolymer-based scaffolds on cell proliferation. In fact, S. Silva et al. tested a chitosan/silk fibroin sponge crosslinked with genipin as a scaffold for cartilage tissue engineering and reported an E’ value of 32.6 kPa, which was found to be favorable for cell proliferation and glycosaminoglycan synthesis by chondrocytes [49]. On the other hand, E. Schuh et al. investigated how matrix elasticity influences the maintenance of chondrogenic activity in chondrocytes cultured on polyacrylamide gel monolayers with varying Young’s moduli (4, 10, 40, and 100 kPa). The results showed good cell proliferation and organized actin structure on scaffolds with moduli greater than 4 kPa. However, higher levels of type II collagen and proteoglycan were observed on the 4 kPa scaffold, suggesting that chondrocyte growth was more stable under these conditions [50]. In addition, Y. Zhou et al. demonstrated that the chondrogenic differentiation of mesenchymal stem cells (MSCs) could be enhanced on soft polyacrylamide hydrogels with a stiffness of 0.5 kPa [51]. M. Murphy et al. investigated the impact of collagen–glycosaminoglycan sponges crosslinked with 1-ethyl-3-(3-dimethylaminopropyl) carbodiimide, which was produced at three different stiffness levels (0.5, 1, and 1.5 kPa). The results revealed SOX 9 expression in the scaffold with the lowest stiffness (0.5 kPa), indicating chondrogenic differentiation of MSCs. In contrast, stiffer scaffolds were associated with an osteogenic lineage [52].

S. Escalante et al. proposed a novel hydrogel composed of chitosan (CHT) and hyaluronic acid (HA), incorporating chondroitin sulfate, which was developed for applications in cartilage repair. An interpenetrating polymer network (IPN) was successfully formed through crosslinking with diisocyanate. The resulting CHT/HA scaffold demonstrated the ability to promote chondrogenic differentiation of mesenchymal stem cells and facilitated cartilage-like tissue formation in vitro. Additionally, this scaffold was anticipated to support the microfracture technique by enhancing clot stabilization and leveraging the mucoadhesive properties of chitosan [19].

The crosslinking reaction considerably influences the stiffness of chitosan-based scaffolds. MDo improved the stiffness of CHT through the formation of a covalent network and showed a synergistic effect when combined with HA. Studies have reported a range of scaffold stiffness values favorable for chondrocyte proliferation. The stiffness of our scaffold falls within this range, suggesting its suitability as a promising scaffold for supporting chondrocyte proliferation and cartilage tissue engineering.

### 2.7. Drug Release Study

**Figure 8:**
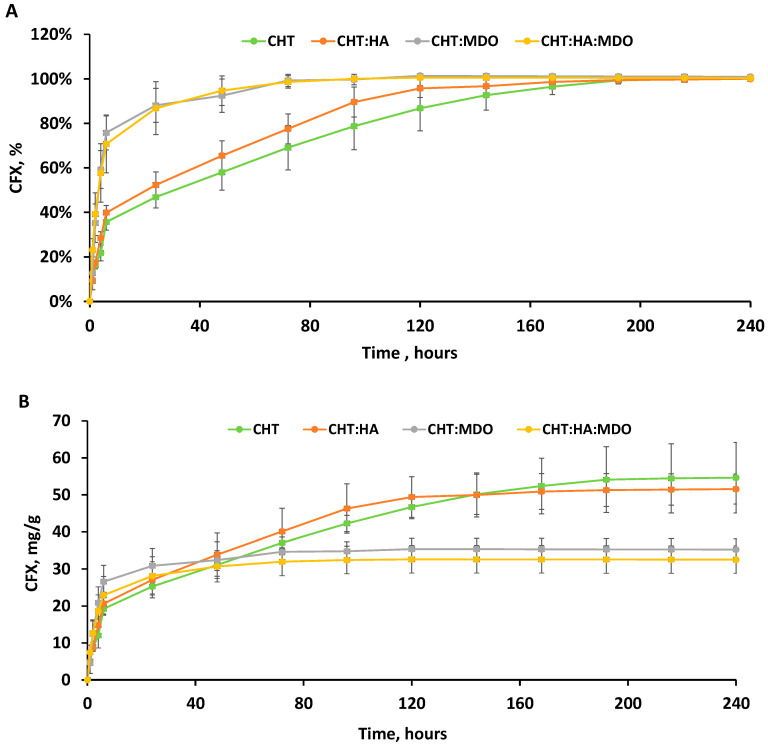
Ciprofloxacin (CFX) release profiles expressed in percentage (%) (**A**), and in quantity (mg/g) (**B**) from CHT, CHT:HA, CHT:MDo, and CHT:HA:MDo. The test was performed under sterile conditions under shaking (80 rpm at 37 °C in triplicate (n = 3)).

Figure 8A,B illustrates the release profiles of ciprofloxacin (CFX) from different sponges, showing both the cumulated amount of CFX expressed in mg/g of sponge and in percentage over time. The percentage of released drug was calculated from the cumulated drug release measured in mg/g after 240 h. All groups showed a pronounced burst release within the first 6 h. Among them, CHT:MDo and CHT:HA:MDo showed the highest release during this period, with 76 ± 8% and 71 ± 13% of the total CFX released, respectively, followed by CHT:HA (40 ± 3%) and CHT (36 ± 4%). After the burst release period of 6 h, a sustained release was observed for non-crosslinked sponges, attaining 100% of released CFX from 192 h, while crosslinked sponges achieved 100% drug liberation after only 72 h.

Both release profiles of the CHT:MDo and CHT:HA:MDo sponges, expressed in mg of CFX (Figure 8B) liberated per gram of scaffold, raised to a plateau corresponding to 34.6 ± 2.5 mg/g and 32.0 ± 3.7 mg/g, respectively. On the other hand, CHT:HA and CHT showed complete release of CFX corresponding to 49.4 ± 5.5 mg/g and 54.5 ± 9.3 mg/g, respectively.

Therefore, non-crosslinked sponges (CHT and CHT:HA) displayed a less important burst effect and a more pronounced sustained drug release than MDo-crosslinked sponges. Interestingly, the presence of HA in crosslinked and non-crosslinked sponges did not modify the release profiles compared with sponges made of CHT only.

The differences in the release kinetics of CFX were triggered by the chemical interactions between sponges and CFX. Indeed, protonated amino groups of CHT are capable of forming electrostatic interactions with CFX. According to A. Semwal et al., electrostatic interactions between the amine groups of CHT and the carboxylate groups of CFX are possible, promoting the affinity between CHT and CFX [53]. The partial conversion of CHT amino groups into imine functions upon crosslinking with MDo is supposed to provoke the decrease of interaction sites with CFX and could explain the lower amount of CFX released by CHT:MDo and CHT:HA:MDo sponges.

Interestingly, our findings suggest that HA did not have a major inhibitory nor enhancing effect on the drug binding and release mechanism, as the plateaus observed in Figure 8B are very similar in the cases of CHT and CHT:HA on the one hand, and CHT:MDo and CHT:HA:MDo on the other hand. Interestingly, comparing the swelling capacities of CHT sponges versus CHT:MDo, a factor of 4.2 is observed (1785% vs. 423%); in parallel, the released CFX measured at 8 days for both these sponges’ groups corresponds to a factor of only 1.57 (54.5 mg CFX/g vs. 34.6 mg CFX/g). The corresponding factors measured for the CHT:HA and CHT:HA:MDo samples are 2.5 and 1.54. These features suggest that the CFX loading capacity of scaffolds cannot be directly related with their swelling capacities. Therefore, the CFX loading capacity of scaffolds depends mainly on physicochemical interactions such as ionic, hydrogen, and van der Waals bonding rather than on the capacity of sponges to absorb the bulk CFX solution. Therefore, as a conclusion, despite their very inferior swelling capacity, the MDo-crosslinked sponges could absorb in the range of 36% less CFX than both the non-crosslinked groups.

### 2.8. Antimicrobial Diffusion Test

The antibacterial activity of the release medium collected from the CFX release kinetics tests were assessed by diffusion tests in the presence of *E. coli*. Aliquots withdrawn within the first 100 h of the release test from the non-crosslinked CHT sponges (CHT and CHT:HA) displayed a stable inhibition diameter in the range of 44 mm that gradually decreased down to 6 mm by the end of the assay (312 h).

**Figure 9:**
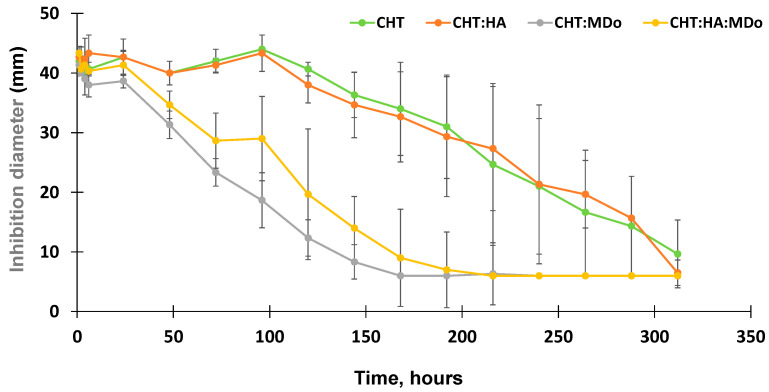
Evolution of the inhibition diameters (from diffusion tests) of supernatants withdrawn from CFX release tests on *E. coli* K12 for CHT, CHT:HA, CHT:MDo, and CHT:HA:MDo sponges (n = 3).

Additionally, the antibacterial activity of the released medium from CHT:MDo showed a continuous decrease of the inhibition diameter from 38.7 ± 1 mm after 24 h, down to 6 mm after 168 h. The same trend was observed for CHT:HA:MDo sponges, with a more gradual decrease in antibacterial activity observed by an inhibition diameter of 41.3 ± 2.3 mm after 24 h progressively decreasing down to 6 mm after 216 h (Figure 9). Consequently, correlations could be observed between the antibacterial test and the release test: (1) the antibacterial activity of the release medium was more sustained in the case of both the CHT and CHT:HA sponges compared with both corresponding MDo-crosslinked sponges; and (2) the HA presence in the sponges did not notably influence the profile of the antibacterial activity.

Similar results were observed in the study of T. Phaechamud et al., where chitosan sponges crosslinked with glutaraldehyde were loaded with doxycycline hyclate, and the antibacterial activity was evaluated against *E. coli* and *S. aureus.* The antibacterial activity was observed in both chitosan and non-crosslinked chitosan sponges; however, the inhibition diameter decreased more rapidly for crosslinked sponges compared to non-crosslinked sponges, and according to the authors, this was due to the rapid release of the drug from the crosslinked sponges. Conversely, the non-crosslinked sponges maintained longer antibacterial activity against *E. coli*, and this was due to the more gradual release profile of the non-crosslinked chitosan sponges [54].

### 2.9. Cytotoxicity Evaluation

**Figure 10:**
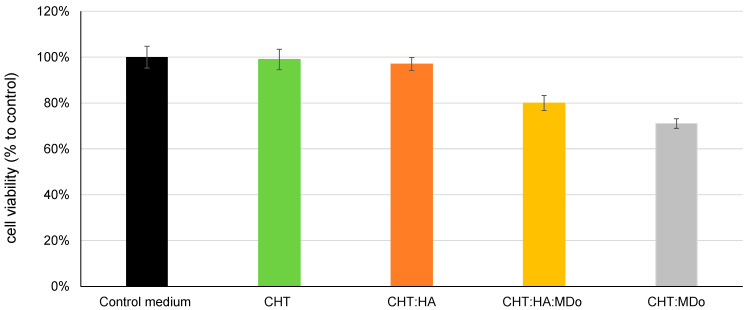
Results of the cytotoxicity study by the extraction method (indirect technique) according to ISO 10993-5 [55], for sponges of CHT, CHT:HA, CHT:MDo, and CHT:HA:MDo.

Figure 10 shows the indirect cytotoxicity test using MC3T3-E1 pre-osteoblastic cells. The results show that CHT and CHT:HA sponges have high viability rates of 100% and 97%, respectively, indicating no toxicity according to the ISO10993-5 standard [55]. The MDo-crosslinked sponges (CHT:MDo and CHT:HA:MDo) showed reduced cell viability, with survival rates of 71% and 80%, respectively. Despite this reduction, the viability remained above the 70% cytotoxicity threshold, indicating that all sponges can be considered non-cytotoxic and displayed safe biological behavior with respect to pre-osteoblastic cells [56,57].

K.W Drzymalska et al. prepared different chitosan films crosslinked by chitosan dialdehyde, starch dialdehyde, and glutaraldehyde. In terms of cytotoxicity, films crosslinked with dialdehyde chitosan displayed the lowest cytotoxicity compared with starch dialdehyde and glutaraldehyde [20]. These findings suggested that chitosan dialdehyde offered not only good results in terms of toxicity, but also in terms of swelling and mechanical and thermal strength, and therefore it represents a promising crosslinking agent for biomacromolecules in biomedical applications.

## 3. Materials and Methods

### 3.1. Materials

Chitosan, with an average molecular weight of 310–375 kDa and a deacetylation degree of 76%, was provided by Sigma Aldrich (Saint-Quentin Fallavier, France). Before use, CHT was milled using a Pulverisette 14 machine (Fritch, Champlan, France), and the powder was then sieved with a mesh size grid of 125 µm (Retsch, Éragny, France). Sodium salt of hyaluronic acid (HA) presented a molecular weight of 1000 kDa (Alfa Aesar Thermo Scientific Chemicals, Illkirch-Graffenstaden, France). Phosphate-buffered saline (PBS), sodium periodate, and lactic acid (LA) were supplied by Sigma Aldrich (Saint-Quentin Fallavier, France), and maltodextrin (Glucidex D19) was provided by Roquette (Lestrem, France). The syringes used for sample preparation had a 5 mL capacity, and were purchased from Terumo (Paris, France).

### 3.2. Methods

#### 3.2.1. Oxidized Maltodextrin Synthesis

Maltodextrin (MD, Glucidex D19^®^) was a gift from Roquette (Lestrem, France). It is a polysaccharide composed of α (1-4)-d-glucose units, derived from the hydrolysis of starch with a dextrose equivalent (DE) of 19 [58]. MD was oxidized according to the protocol of Thomas P. Clarck et al. [59]. Briefly, 41.03 g of MD were dissolved in deionized water, and 56.40 g of sodium periodate (NaIO_4_) were added under a nitrogen (N_2_) atmosphere. The molar ratio of glucopyranose repeat units of MD vs. NaIO_4_ in the reaction vessel was 0.96. The reaction was carried out under stirring for 24 h and protected from light. The reaction mixture was passed through a column containing an ion-exchange resin (Amberlite™ IRN150, Thermo Fisher Scientific, Asnières-sur-Seine, France) for NaIO_4_ removal. Finally, the purified solution was freeze-dried at −55 °C for 24 h (Christ Alpha 1-2 LD, Grosseron, Couëron, France) to produce MDo powder. The oxidation degree was determined by acid–base titration according to S. Veelaert et al. [60], and was estimated at 60%.

#### 3.2.2. Hydrogel and Sponge Preparation

In the first stage, the CHT and CHT:HA powders (ratio 5:1) were milled or co-milled using a Retsch MM 400 (Verder SARL, Eragny sur Oise, France) to obtain homogeneous powder mixtures; further details on the formulations used are found in Table 2.

**Figure 11:**
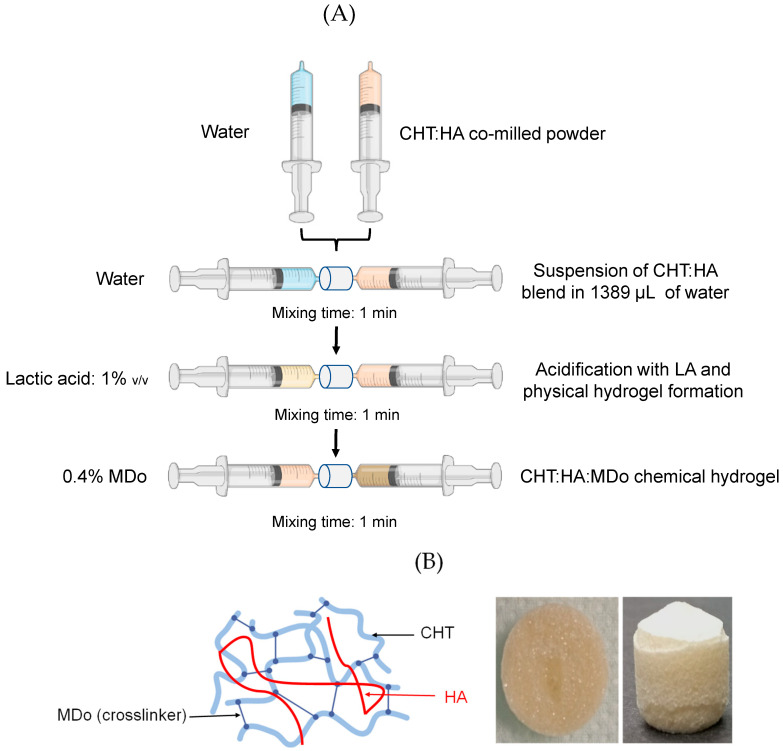
(**A**) Process steps for preparing chemical hydrogels of chitosan (CHT) and hyaluronic acid (HA) crosslinked with oxidized maltodextrin (MDo). (**B**) Simplified representation of the semi-interpenetrated network consisting of HA entangled in the crosslinked CHT:MDo network, and the image of the sponge after freeze-drying the CHT:MDo hydrogel.

Hydrogels were prepared using an interconnected syringe setup [38] (Figure 11). In the first step, the first syringe containing the powder (CHT or CHT:HA mixture) was connected to a second syringe containing ultrapure water (total volume = 1.5 mL), and the syringe connection was performed with a female–female Luer lock connector. The syringe preparation method consisted of alternately pushing both plungers for one minute, and then a suspension was obtained. The weights of the CHT and HA powders introduced in the syringes were adjusted in order to reach concentrations of 5% for CHT and 1% for HA.

In the second step, 15 µL of lactic acid (LA) were mixed with the suspensions. The suspensions were rapidly transformed into a viscous solution (from CHT alone) or into CHT:HA physical hydrogels. The volume of LA was adjusted in order to reach the concentration of 1% *v*/*v* in the final solution or hydrogel.

In the third step, the CHT solution and CHT:HA hydrogel were chemically crosslinked with MDo. The weight of MDo was adjusted in order to reach a concentration of 0.4% *w*/*v* in the hydrogel. Formulations are reported as X:Y:Z where X corresponds to the concentration of CHT (fixed at 5%), Y corresponds to the concentration of HA (fixed at 0 or 1%), and Z corresponds to the concentration of MDo (fixed at 0 or 0.4%).

In the fourth step, the hydrated formulations were left in their preparation syringes and frozen at −21 °C for 12 h, and freeze-dried at constant parameters of 0.06 mbar and −53 °C for 48 h. Finally, scaffolds were obtained in the shape of cylinders of 12 mm diameter and 14 mm height, presenting an open porous structure like a sponge (Figure 1). Samples containing MDo were left at ambient temperature for 2 h before freezing and lyophilization in order to allow the crosslinking reaction to occur.

#### 3.2.3. FTIR Analysis

Sponges were analyzed using an ATR-FTIR Spectrum100 (Perkin-Elmer, Villebon-sur-Yvette, France) and data acquisition was carried out using the Spectrum software OMNIC. An accumulation of 32 scans in a spectral range from 4000 to 600 cm^−1^ was carried out for each sample, with a resolution of 2 cm^−1^.

#### 3.2.4. Swelling Test

Swelling studies of the sponges were performed in a phosphate-buffered saline (PBS) medium (pH = 7.4). Dry sponges (27 ± 5 mg) were weighed and placed in a flask containing 10 mL of PBS. The flasks were placed in an incubator (Gerhard, Paris, France) at 37 °C under agitation (80 rpm). The incubation times were 2 h, 4 h, 6 h, and 24 h. Sponges were removed from the batches, and the excess PBS solution was removed with absorbent paper [61]. The mass of the wet sponges was then measured using a precision balance with a precision of ±4 × 10^−4^ g (Kern, GmbH, Großmaischeid, Germany). Experiments were performed in triplicate (n = 3) for each time point. The swelling ratio was determined according to the following Formula (1):

(1) $$Swelling\;\left(\%\right)=\frac{W_{s}-W_{0}}{W_{0}}\times 100\;\;$$

where W_0_ = initial weight of the dry sponge, and W_s_ = weight of the swollen sponge at time t. At the end of the test, the wet sponges were oven-dried at 60 °C to estimate the rate of dry mass loss after sponge swelling, and the values of the swelling rates and dry mass after 24 h of swelling in PBS (37 °C, 80 rpm) were analyzed.

#### 3.2.5. Dynamic Vapor Sorption (DVS)

The hygroscopic behavior of the sponges was measured gravimetrically using a TGA Q5000 SA (TA Instruments, Guyancourt, France). The instrument consists of a microbalance and a humidity–temperature-controlled chamber. Relative humidity (RH) was controlled by a N_2_ alpha gas flow, dry (RH = 0%) or water-saturated (RH = 95%) (global flow set to 200 mL/min), in the chamber [62].

The sample (5 mg) was placed in the chamber. The water adsorption isotherm was obtained after a preliminary drying at 60 °C (time ≤ 3 h) and 0% RH. This step was maintained until the weight stabilized and afterward, the temperature started to decrease down to stabilize at 25 °C. Then, the relative humidity increased progressively up to 95% RH. RH was increased by steps of 5% in the chamber once the weight of the sample was constant, by 0.01% for 5 min. The water desorption isotherm was obtained by decreasing RH down to 0%.

#### 3.2.6. In Vitro Degradation Study of Sponges

The degradation of the sponges was evaluated under hydrolytic, enzymatic, and acidic conditions (detailed hereafter) and was calculated as the residual weight of the sponges expressed in percent using the following Formula (2) at each time point of the experiment, between 1 and 90 days:

(2) $$Remaining\;weight\;\left(\%\right)=\left[1-\frac{W_{0}-W_{t}}{W_{0}}\right]\times 100$$

where W_0_ = initial weight of the dry sponge, and W_t_ = weight of the dried sponge after degradation at time t.

##### Hydrolytic Degradation

Dry sponges were immersed in 10 mL of filtered PBS (pH 7.4) and incubated at 37 °C under agitation (80 rpm). Samples were collected at 1, 2, 7, 14, 21, 40, 60, and 90 days. At each time point, the sponges were rinsed with distilled water to remove salts, dried at 60 °C for 12 h, and weighed.

##### Enzymatic Degradation

A lysozyme solution (75,579 U/mg, Fluka, Waltham, MA, USA) was prepared at a concentration of 0.5 mg/mL in filtered PBS (pH 7.4) [63]. Sponges were incubated in 10 mL of this solution at 37 °C under agitation (80 rpm), with a renewal of the solution every 72 h. Samples were collected at the same time points as for hydrolytic degradation, rinsed, and dried [43].

##### Degradation in Acidic Medium

Sponges were incubated in 10 mL of a 1% *v*/*v* lactic acid solution (pH = 2.3) at 37 °C under agitation (80 rpm) for 7 days. After incubation, the samples were rinsed, dried at 60 °C for 12 h, and weighed.

#### 3.2.7. SEM Analysis

Morphological observation of the different sponges was carried out using scanning electron microscopy (SEM) (Hitachi Flex 1000, Tokyo, Japan), and the acceleration voltage was 5 kV. All samples were sputtered with chromium.

The pore diameters were measured using the SEM Flex 1000 software (FlexSEM 1000 II VP-SEM), and the average pore size was determined by taking 20 measurements for each sample.

#### 3.2.8. Dynamic Mechanical Analysis (DMA)

The mechanical properties of the different sponges in the swelled state were characterized by dynamical mechanical analysis using RSA III rheometric equipment (TA instruments, Guyancourt, France). The principle of the test is to apply a sinusoidal strain ε = ε_o_ sin (2πft) and measure the stress response, which is also sinusoidal in the linear viscoelastic domain, with a phase shift between 0 and 90° related to the viscoelastic response of the material. The storage modulus E’ (which is the part of the response in phase with the solicitation) corresponds to the stiffness of the material, while the loss modulus E’’ (which is the part of the response with a 90° phase shift with the solicitation) corresponds to the viscous part of the material.

In this study, tests were performed in compressive mode at 37 °C on cylindrical samples of 10 mm height and 12 mm diameter. Before the measurements, sponges were immersed for 24 h in a PBS solution at 37 °C.

Two kinds of experiments have been performed: a strain sweep test in which an increasing strain amplitude ε_o_ from 0.1% to 10% at a constant frequency of 1 Hz was applied to determine the linear viscoelastic domain of the sponges, and a dynamic frequency sweep study from 0.1 to 10 Hz at a constant strain amplitude of 1% to simulate the physiological conditions found in cartilage.

#### 3.2.9. Drug Release Study

CHT-based sponges (diameter 8 mm, height 5 mm) were weighted and UV-decontaminated for 30 min under a germicide UV lamp (254 nm). They were immersed in 10 mL of ciprofloxacin hydrochloride solution (CFX, Fagron, Rotterdam, the Netherlands) at 2 mg/mL for 4 h under agitation (80 rpm). The sponges were stored at 37 °C for 12 h in a sterile culture dish (24 wells). A total of 1 mL of sterile PBS was used as the release medium, which was completely replaced after 1 h, 2 h, 4 h, 6 h, and every 24 h over 10 days. The test was carried out at 37 °C and under stirring (80 rpm). The quantification of ciprofloxacin was performed using a Shimadzu LC2040C HPLC, Nexera i (Shimadzu, Kyoto, Japan), equipped with a UV detector). Samples were eluted on a Gemini-NX C18 column, 250 × 4.6 mm, 5 µm (Phenomenex, Le Pecq, France). A total of 20 µL of release medium was injected into a mobile phase consisting of a mixture of 0.01 M KH_2_PO_4_ buffer, pH = 3/acetonitrile, 82/18, *v*/*v*, circulated at a flow rate of 0.8 mL/min. Detection was at 278 nm and chromatograms were processed using the LabSolution software (Shimadzu, Kyoto, Japan). All manipulations were performed in the microbiological safety post in triplicate (n = 3).

#### 3.2.10. Antibacterial Activity Assay

In parallel with the drug release study, the antibacterial activity of ciprofloxacin released in the supernatant by the sponges at each time was determined. Briefly, Petri dishes were prepared with 18 mL of Mueller–Hinton agar (MHA) (ThermoScientific, Oxiod29 Microbiological Products, Asnières-sur-Seine, France). *E. coli* K12 was suspended in 10 mL of cysteine-containing Ringer’s solution to form a stock suspension with a density of 10^4^ CFU/mL. Each agar plate was inoculated homogeneously with 0.1 mL of the bacterial suspension, and 6 mm wells were created in the culture gel where 50 µL of the release medium were introduced. All plates were incubated at 37 °C for 24 h. The inhibition diameter formed around the wells were measured in mm as a function of time of contact of the sponges in the release media. The tests were carried out in triplicate (n = 3).

#### 3.2.11. Cytotoxicity Study

The cytotoxicity of the sponges was assessed by the extraction method using MC3T3-E1 pre-osteoblastic cells (ATCC^®^ CRL 2593™, Manassas, VA, USA) according to ISO 10993-5 [55]. The sponges were previously swollen in 20 mL of complete culture medium (CCM) per sponge for 5 h at 37 °C under shaking (80 rpm), rinsed with distilled water, and weighted. A total of 200 mg of wet mass of the sponge was taken and immersed in 1 mL of complete culture medium (CCM) consisting of α-MEM (Gibco, Asnières-sur-Seine, France) and 10% fetal bovine serum. The mixture was placed in an incubator (New Brunswick Scientific, Innova 40, Edison, NJ, USA) for 24 h at 37 °C under agitation (80 rpm).

At the same time, the MC3T3-E1 pre-osteoblastic cells were seeded in a 96-well plate at a density of 4 × 10^3^ cells per well for 24 h to establish a monolayer. After incubation of the sponges, the extraction medium was filtered (0.22 µm) and 100 µL of this sterile extraction medium were used to replace the medium in each well. The cells were cultured in this medium for 24 h in an incubator (MIDI 40 CO_2_ incubator, Thermo Fisher Scientific, France) at 37 °C in a 5% CO_2_ atmosphere.

After incubation, 200 µL of Alamar^®^ Blue reagent (Thermo Fisher Scientific, France) were added to each well and incubated for 2 h at 37 °C. After this period, 150 µL from each well were removed for fluorescence analysis. Fluorescence intensity was measured using a fluorometer (CLARIOstar^®^, BMG Labtech, Ortenberg, Germany) with an excitation wavelength of 530 nm and an emission wavelength of 590 nm. Cell viability was determined by expressing the fluorescence intensity as a percentage of the control group.

## 4. Conclusions

Spongy scaffolds designed for cartilage regeneration were obtained from freeze-drying CHT-based solutions also containing HA and MDo as a crosslinking agent. The resulting materials (CHT, CHT:HA, CHT:MDo, and CHT:HA:MDo) analyzed by SEM presented an alveolar structure with thin walls and a pore size in the range of 200 µm, which is favorable toward cell colonization. After swelling in PBS solution and a drying cycle, CHT and CHT:HA samples displayed pronounced residual deformation (increase of pore size), contrary to the corresponding MDo-crosslinked sponges. In parallel, MDo crosslinking substantially limited sponge swelling in PBS compared with non-crosslinked sponges. The DVS study displayed a slight reduction of the hygroscopic character of sponges in water vapor-saturated ambience. All sponge groups presented the same range of stability in PBS over 90 days. However, in the presence of lysozyme, non-crosslinked sponges were rapidly fully degraded within this period, contrary to crosslinked ones. Crosslinking also prevented scaffold degradation in the acidic medium. The DMA study on swelled scaffolds showed that HA and MDo greatly increased the storage modulus, from 29 kPa up to 197 kPa. Sponges were then loaded with CFX and tested for the assessment of their drug release profiles. It was observed that the CFX loading capacity of sponges was promoted by CHT–CFX interactions but was not greatly influenced by HA’s presence. Therefore, despite a far lesser swelling capacity, crosslinked sponges could absorb only 36% less of the drug than non-crosslinked sponges. This revealed specific drug–CHT interactions by ionic, hydrogen, and van der Waals bonding that were slightly reduced under the decrease of amino groups of CHT converted into imine crosslinks. MDo-treated sponges displayed faster CFX release profiles in batch, supported by microbiology tests showing more prolonged antimicrobial activity of non-crosslinked groups against *E. coli*. Crosslinking with MDo did not affect the biological response of cells, suggesting that these scaffolds are suitable for applications in biological tissues and are promising candidates for cartilage tissue engineering.

### Statistical Analysis of Results

All experiments were conducted in triplicate (n = 3), and data are presented as mean ± standard deviation. As this study represents an early-stage investigation focused on material development and characterization, formal statistical analyses were not performed due to the limited sample size. The primary objective was to identify consistent trends and demonstrate the feasibility of the scaffold formulation, which was confirmed through multiple independent experiments. We acknowledge the importance of larger sample sizes and comprehensive statistical analysis for future work.

## Figures and Tables

**Table 1:** Hydrogel formulations. CHT: chitosan; HA: hyaluronic acid; LA: lactic acid; MDo: oxidized maltodextrin (volume of water = 1.5 mL).

CHT:HA:MDo	% CHT (*w*/*v*)	% HA (*w*/*v*)	% LA (*v*/*v*)	% MDo (*w*/*v*)
5:0:0	5	0	1	0
5:0:0.4	5	0	1	0.4
5:1:0	5	1	1	0
5:1:0.4	5	1	1	0.4

**Table 2:** Pore mean diameters (in µm) of sponges based on CHT, CHT:HA, CHT:MDo, and CHT:HA:MDo after preparation and after swelling in PBS and freeze-drying. Values of swelling rates (according to Equation (1)) and remaining dry weight (according to Equation (2)) were measured after 24 h of swelling in PBS (37 °C, 80 rpm). Moisture content (g of water/g of sample) at 95% of relative humidity (HR) was measured by DVS.

Sponge	Pore Size (µm) Before Swelling	Pore Size (µm) After Swelling	Swelling Rate After 24 h of Incubation	Remaining Dry Weight After 24 h of Swelling	Moisture Content (g/g) at 95% HR
CHT	182.7 ± 44.1	298.4 ± 123	1785 ± 119%	91.1 ± 3.4%	0.85
CHT:HA	190.5 ± 71	243.5 ± 143	1480 ± 167%	86.7 ± 1.7%	0.85
CHT:MDo	215.0 ± 93.5	220.6 ± 53.4	423 ± 47%	88.7 ± 0.3%	0.78
CHT:HA:MDo	204.0 ± 67	213.0 ± 111	587 ± 11%	89.7 ± 0.54%	0.77

## Data Availability

Data is contained within the article.
